# Time series analysis of reported cases of hand, foot, and mouth disease from 2010 to 2013 in Wuhan, China

**DOI:** 10.1186/s12879-015-1233-0

**Published:** 2015-11-03

**Authors:** Banghua Chen, Ayako Sumi, Shin’ichi Toyoda, Quan Hu, Dunjin Zhou, Keiji Mise, Junchan Zhao, Nobumichi Kobayashi

**Affiliations:** Department of Infectious Diseases Prevention and Control, Wuhan Centers for Disease Control and Prevention, Wuhan, Hubei China; Department of Hygiene, Sapporo Medical University School of Medicine, S-1, W-17, Chuo-ku, Sapporo, 060-8556 Hokkaido Japan; Department of Information Engineering, College of Industrial Technology, Hyogo, Japan; Wuhan Centers for Disease Control and Prevention, 24 Jianghanbei Road, Wuhan, 430000 Hubei China; Department of Admission, Center of Medical Education, Sapporo Medical University, Hokkaido, Japan; School of Mathematics and Statistics, Hunan University of Commerce, Changsha, Hunan China

**Keywords:** Hand, foot, and mouth disease, Seasonality, Meteorological variable, Time series analysis, Spectral analysis

## Abstract

**Background:**

Hand, foot, and mouth disease (HFMD) is an infectious disease caused by a group of enteroviruses, including Coxsackievirus A16 (CVA16) and Enterovirus A71 (EV-A71). In recent decades, Asian countries have experienced frequent and widespread HFMD outbreaks, with deaths predominantly among children. In several Asian countries, epidemics usually peak in the late spring/early summer, with a second small peak in late autumn/early winter. We investigated the possible underlying association between the seasonality of HFMD epidemics and meteorological variables, which could improve our ability to predict HFMD epidemics.

**Methods:**

We used a time series analysis composed of a spectral analysis based on the maximum entropy method (MEM) in the frequency domain and the nonlinear least squares method in the time domain. The time series analysis was applied to three kinds of monthly time series data collected in Wuhan, China, where high-quality surveillance data for HFMD have been collected: (i) reported cases of HFMD, (ii) reported cases of EV-A71 and CVA16 detected in HFMD patients, and (iii) meteorological variables.

**Results:**

In the power spectral densities for HFMD and EV-A71, the dominant spectral lines were observed at frequency positions corresponding to 1-year and 6-month cycles. The optimum least squares fitting (LSF) curves calculated for the 1-year and 6-month cycles reproduced the bimodal cycles that were clearly observed in the HFMD and EV-A71 data. The peak months on the LSF curves for the HFMD data were consistent with those for the EV-A71 data. The risk of infection was relatively high at 10 °C ≤ *t* < 15 °C (*t*, temperature [°C]) and 15 °C ≤ *t* < 20 °C, and peaked at 20 °C ≤ *t* < 25 °C.

**Conclusion:**

In this study, the HFMD infections occurring in Wuhan showed two seasonal peaks, in summer (June) and winter (November or December). The results obtained with a time series analysis suggest that the bimodal seasonal peaks in HFMD epidemics are attributable to EV-A71 epidemics. Our results suggest that controlling the spread of EV-A71 infections when the temperature is approximately 20–25 °C should be considered to prevent HFMD infections in Wuhan, China.

**Electronic supplementary material:**

The online version of this article (doi:10.1186/s12879-015-1233-0) contains supplementary material, which is available to authorized users.

## Background

Hand, foot, and mouth disease (HFMD) is an infectious disease that typically presents as vesicular exanthema of the oral mucosa and peripheral extremities. Enteroviruses, such as Coxsackievirus A16 (CVA16) and Enterovirus A71 (EV-A71), are most commonly isolated from HFMD patients [1]. Over the past decade, Asian countries have experienced enormous large-scale HFMD outbreaks, with deaths predominantly among children [2–6]. The epidemics in China have been particularly serious and HFMD has become one of the leading causes of child death in China and a public health priority [7]. In 2008–2012, 7,200,092 cases of HFMD, including 2457 fatal cases, were reported by the Chinese Center for Disease Control and Prevention [8]. However, no vaccine or effective curative treatment is currently available. The incidence of HFMD will be also significantly affected by the continued mutation of the virus and increasing climate change. Therefore, HFMD remains an important public health problem in China.

Many studies have reported the seasonality of HFMD epidemics in China, and understanding the seasonality of these epidemics may identify potentially modifiable risk factors. Epidemics in several regions of China peak in late spring/early summer, with a second smaller peak in late autumn/early winter [9–13]. Researchers have interpreted the seasonality of HFMD cases in terms of climate variables in specific regions. Meteorological parameters, such as temperature and relative humidity, may affect the transmission and frequency of HFMD. However, the effects of climate variables are not consistent across published studies, and these discrepancies could arise from various local climatic conditions, differences in socioeconomic status, and the demographic characteristics of different regions. Therefore, our understanding of the impact of seasonal and meteorological variables on disease transmission remains limited. Further research is required into the effects of climate variations on the incidence of HFMD.

Wuhan in Hubei Province is the largest mega-city in Central China, and has experienced a relatively high prevalence of HFMD in recent years. A better understanding of the temporal pattern of HFMD incidence might allow the appropriate allocation of health-care resources for better disease control and prevention. No study has yet examined the effects of meteorological variables on the occurrence of HFMD in Wuhan.

In this study, we investigated the association between the incidence of HFMD and its pathogens and several meteorological variables (including monthly average temperature, maximum temperature, minimum temperature, relative humidity, total rainfall, and wind velocity) in Wuhan, China, where high-quality surveillance data for HFMD have been collected. We used the time series analysis method “MemCalc” (Suwa-Trast, Tokyo, Japan) [14–16], which has been successfully used to investigate associations between the occurrence of infectious diseases, pathogens, and meteorological variables, including rotavirus in India [15], cholera in Bangladesh [17], and chickenpox in Japan [18]. Based on the result for the seasonality of HFMD, we conducted a prediction analysis for HFMD epidemics.

## Methods

### Study area

**Fig. 1:**
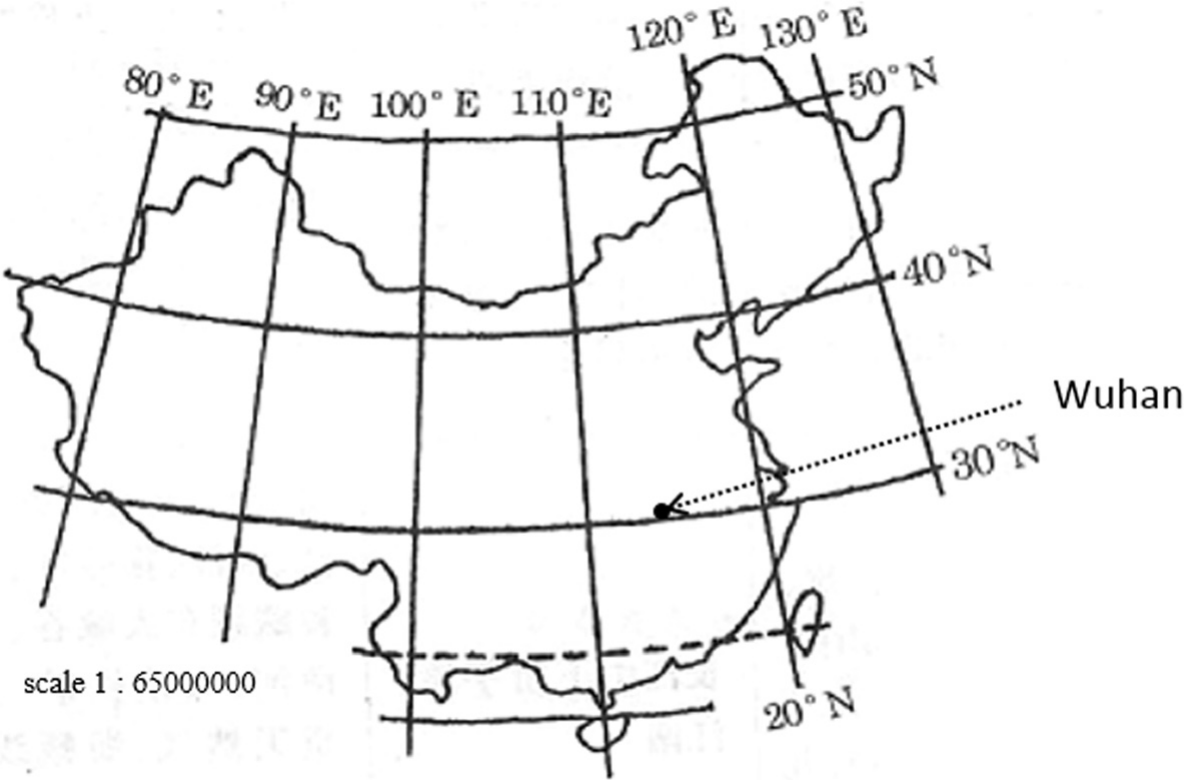
Location of Wuhan in China. Source: revised from “Chinese latitude and longitude map” (http://www.baidu.com)

Figure 1 shows the location of Wuhan, China. Wuhan, the capital city of Hubei Province in central China, has a total area of 8494 km^2^ and a population of 10.3 million. Wuhan is situated at a latitude of 30°34′N and a longitude of 114°16′E, in an area with a subtropical wet monsoonal climate, where the four seasons are very clearly defined. Based on the assumption that the seasons coincide with the weather and temperature patterns in Wuhan, the seasons were defined as spring (April), summer (May–September), autumn (October), and winter (November–March). The monsoon occurs in Wuhan from the middle of June to the middle of July (summer) every year.

### Data

#### HFMD data

A probable case of HFMD is defined as a patient with papular or vesicular rash on the hands, feet, mouth, or buttocks, with or without fever. A confirmed case is defined as a probable case with laboratory evidence of enteroviral infection (including EV-A71, CVA16, or other non-EV-A71 or non-CVA16 enteroviruses) detected with reverse transcription—polymerase chain reaction (RT-PCR), real-time RT-PCR, or viral isolation [19]. Probable and confirmed cases are reported on-line to the China Information System for Disease Control and Prevention (CISDCP, http://www.cdpc.chinacdc.cn) by all the hospitals in Wuhan, using a standardized form. In this study, we analyzed the daily number of cases of HFMD reported in Wuhan between January 1, 2010 and December 31, 2014 (1825 data points). The data are available from the CISDCP website through the Wuhan Centers for Disease Control and Prevention. First, we investigated the associations between the incidence of HFMD and its pathogens and meteorological variables, using the HFMD data from between January 2010 and June 2013 (1276 data points), and then we conducted a prediction analysis using the HFMD data from between July 2013 and December 2014 (549 data points).

#### Pathogen data

According to the national guidelines [20], the samples were collected from the first five probable cases who presented to hospital outpatient departments each month in each of the 13 districts of Wuhan. The appropriate clinical specimens, including throat swabs, rectal swabs, fecal samples, vesicular fluid, and/or cerebrospinal fluid, were collected. The samples were identified with real-time PCR in biosafety level 2 facilities in the Wuhan Center for Disease Control and Prevention. The test results were classified into four categories: enterovirus negative, EV-A71 positive, CVA16 positive, or positive for another enterovirus without further serotype identification. All pathogen data were uploaded to CISDCP and were downloaded as monthly data. We accessed the relevant pathogen data for the study period from January 2010 and June 2013 (42 data points) from the CISDCP website and the case data for HFMD.

#### Meteorological data

Daily meteorological data, including average temperature, maximum temperature, minimum temperature, relative humidity, total rainfall, and wind velocity, were collected in the study region by the Meteorological Department, Wuhan, which received and managed real-time data from 116 meteorological surveillance sites widely distributed in Wuhan. The daily data were gathered for 1276 days from January 2010 to June 2013 (1276 data points).

The descriptive statistics for the monthly meteorological data are shown in Table 1. The mean monthly average values in Wuhan were: temperature 16.5 °C, maximum temperature 21.4 °C, minimum temperature 12.6 °C, relative humidity 78.2 %, total rainfall 111.3 mm, and wind velocity 2.1 m/s.

**Table 1:** Summary statistics for the monthly meteorological conditions in Wuhan, China

Variable	Minimum	Median	Mean	Maximum	SD
T{A} (°C)	1.6	16.9	16.5	30.0	9.1
T{M} (°C)	6.3	22.4	21.4	34.2	8.9
T{m} (°C)	−1.9	12.8	12.6	26.9	9.2
RH (%)	64.0	79.2	78.2	85.9	6.1
RF (mm)	7.2	90.2	111.3	384.4	99.1
WV (m/s)	1.3	2.0	2.1	2.9	0.3

### Time series analysis

The series of analyses used in the present study was composed of spectral analyses based on the maximum entropy method (MEM) in the frequency domain and the nonlinear least squares method (LSM) in the time domain. This method of analysis can be used for prediction analysis [21, 22].

#### *Theoretical background* [21]

We assumed that the time series data *x*(*t*) (where *t* = time) were composed of systematic and fluctuating parts [23]:

1 $$ x(t) = \mathrm{systematic}\ \mathrm{part} + \mathrm{fluctuating}\ \mathrm{part}. $$

The systematic part in Eq. (1) is regarded as the underlying variation in the original time series, and the fluctuating part, including undeterministic components such as noise, was obtained as the residual time series when the underlying part was subtracted from the original time series. The estimation of the underlying variation is a key point.

The underlying variation in the original time series data *x*(*t*) is assumed to be described by the function *X* (*t*), as follows:

2 $$ X(t)={A}_0+{\displaystyle \sum_{n=1}^{N_p}{A}_n \cos \left\{2\pi {f}_n\left(t+{\theta}_n\right)\right\},} $$

which is calculated using the LSM for *x*(*t*) with unknown parameters *f*_*n*_, *A*_0_, and *A*_n_ (*n* = 1, 2, 3, …, *N*), where *f*_*n*_ (=1/*T*_*n*_; *T*_*n*_ is the period) is the frequency of the *n*th component; *A*_0_ is a constant that indicates the average value of the time series data; *A*_*n*_ and *θ*_*n*_ are the amplitude and the phase of the *n*th component, respectively; and *N*_*p*_ is the total number of components. The LSM using Eq. (2) must be nonlinear. Linearization of this nonlinearity is required to obtain the unique optimum values of these parameters. In the present study, linearization was achieved using the value of *f*_*n*_ estimated with the MEM spectral analysis.

An outline of the analysis procedure is described as follows. The details of the procedure for the method are described in our previous work [14, 22].

1. *Setting up the time series data for analysis.* The sampling intervals for the HFMD and meteorological data (daily) and pathogen data (monthly) differed. To analyze these three kinds of data together, it was necessary to choose equal sampling time intervals. Therefore, we calculated the monthly data for the HFMD cases and meteorological variables (42 data points) from the original daily data to conform to the monthly pathogen data. All the meteorological parameters studied and the values used for testing the associations are summarized in Additional file 1. For example, the monthly average maximum temperature was calculated by averaging the daily maximum temperature for a month, and the total rainfall was calculated by summing the amount of rainfall measured for the whole month. The monthly meteorological variables are described as follows: T{A}, average temperature (°C); T{M}, maximum temperature (°C); T{m}, minimum temperature (°C); RH, relative humidity (%); RF, total rainfall (mm); and WV, wind velocity (m/s).
2. *Determination of T*_*n*_*(spectral analysis).* The value of *T*_*n*_ was determined from the positions of the peaks in the MEM power spectral density (MEM-PSD). The MEM spectral analysis has a high degree of resolution and is useful for clarifying periodicities within short time series, such as the time series data examined in this study [21]. The MEM spectral analysis produces a power spectral density (PSD). The formulation of the MEM-PSD is described in an additional file (see Additional file 2).
3. *Determination of N*_*p*_*(assignment of the dominant periodic modes).* The contribution of the dominant periodic modes to the underlying variation can be estimated easily from the trend in the standard deviations (SD) of the residual time series *x*_R_(*t*) (= *x*(*t*) – *X*(*t*)). The value of *N*_*p*_ is then determined.
4. *Determination of A*_*0*_*, A*_*n*_, *and θ*_*n*_*(least-squares analysis).* The optimum values for parameters *A*_0_, *A*_*n*_, and *θ*_*n*_ (*n* = 1, 2, 3, …, *N*_*p*_), in Eq. (2), but not *N*_*p*_, were determined exactly from the optimum least squares fitting (LSF) curve calculated using the periodic function (Eq. (2)) with the MEM-estimated periods (*T*_*n*_).
5. *Prediction analysis.* The optimum LSF curve *X*(*t*) was extrapolated to predict the original time series because the optimum LSF curve is regarded as the predictable part [24]. For the HFMD data, we extended *X*(*t*) from the analysis range (January 2010–June 2013) to the prediction range (July 2013–December 2014).

### Statistical calculations

All statistical analyses were performed with SPSS 17.0 J for Windows (SPSS Inc., Chicago, IL, USA), and Spearman’s rank correlation (*ρ*) was used. A two-tailed analysis was used for all statistical tests and a *p* value of ≤ 0.05 was considered the criterion for statistical significance.

### Correlation between pathogen data and meteorological data

The average occurrence of EV-A71 infections and CVA16 infections in the different domains of average temperature (T{A}), *T* to *T* + *ΔT,* was calculated with the following formula [25]:

3 $$ {N}_{T\left\{A\right\},j}=\frac{{\displaystyle \sum_i^n{C}_{i,j}f\left({t}_i\right)}}{{\displaystyle \sum_i^nf\left({t}_i\right)}},\ j=\left\{\begin{array}{l}E:EV-A71\\ {}C:CVA16\end{array}\right. $$

where *i* is a sequence from 0 to *n*, *t*_*i*_ is T{A} for the *i*th month period, *C*_*i,j*_ is the total number of cases of pathogen *j* infection in the *i*th month, and *f*(*t*_*i*_) is a function with the following values:

4 $$ f\left({t}_i\right)\left\{\begin{array}{l}=1\ \mathrm{when}\ T\le {t}_i<T+\varDelta T\\ {}=0\ \mathrm{otherwise}\end{array}\right. $$

The numerator on the right side of Eq. (3) represents the sum of all C_*i,j*_ comprising the 1-month average temperature (*t*_*i*_) within the temperature domain of *T* to *T* + *ΔT* during the data period. The denominator is the total number of occasions upon which *T* < *t*_*i*_ < *T* + *ΔT* during the same data period.

Similarly, the average occurrences of pathogen infections in the different variable domains for maximum temperature (*N*_T{M},E_ and N_T{M},C_), minimum temperature (*N*_T{m},E_ and N_T{m},C_), relative humidity (*N*_RH,E_ and *N*_RH,C_), total rainfall (*N*_RF,E_ and *N*_RF,C_), and wind velocity (*N*_WV,E_*and N*_WV,C_) were determined. The variables *t*_*i*_, *T*, and *ΔT* for T{A} in Eq. (4) were replaced with *tM*_*i*_, *TM*, and *ΔTM*, respectively, for T{M}; with *tm*_*i*_, *Tm*, and *ΔTm*, respectively, for T{m}; with *h*_*i*_, *H*, and *ΔH*, respectively, for RH; with *r*_*i*_, *R*, and *ΔR*, respectively, for RF; and with *w*_*i*_, *W*, and *ΔW*, respectively, for WV.

**Fig. 2:**
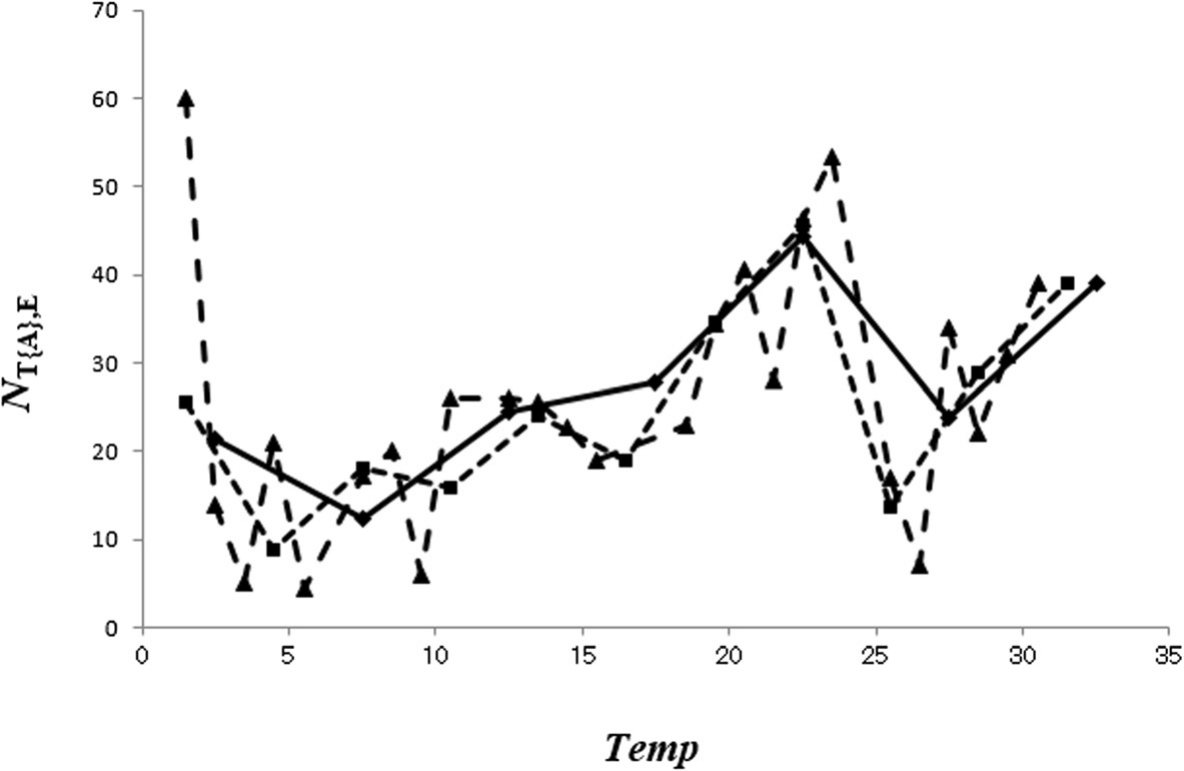
Dependence of the occurrence of EV-A71 infection and average temperature (*N*_T{A},E_) on temperature interval (*ΔT*). Dashed line, *ΔT* = 1 °C; dotted line, *ΔT* = 3 °C; solid line, *ΔT* = 5 °C

In Fig. 2, we show the values for *N*_T{A},E_ against *Temp (Temp*; temperature [°C]) when *ΔT* = 1, 3, and 5 °C. When *ΔT* = 1 °C and 3 °C, the curve of *N*_T{A},E_ displays irregular variability, whereas when *ΔT* = 5 °C, the curve of *N*_T{A},E_ becomes regular in shape. Therefore, we used *ΔT* = 5 °C for T{A} in the present study. Similarly, the values for *ΔTM, ΔTm*, *ΔH*, *ΔF*, and *ΔW* were determined as 5 °C, 5 °C, 5 %, 30 mm, and 0.1 m/s, respectively.

## Results

### Case description

**Fig. 3:**
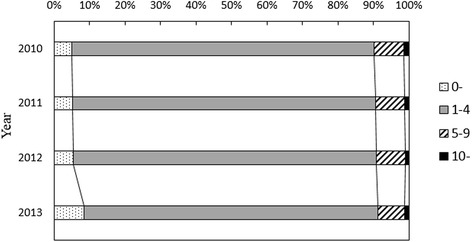
Age distribution of the reported HFMD cases at hospitals in the whole of Wuhan

From January 2010 to June 2013, 48,882 cases of HFMD were reported to the CISDCP, 4.5 % (2195 HFMD cases) of which were laboratory-confirmed. The age distribution of the reported cases is shown in Fig. 3. The number of reported cases varied greatly with age, with the highest proportion in children under 5 years. This age group contributed over 90 % of the reported cases during the study period.

### Temporal variations in HFMD data, pathogen data, and meteorological data

**Fig. 4:**
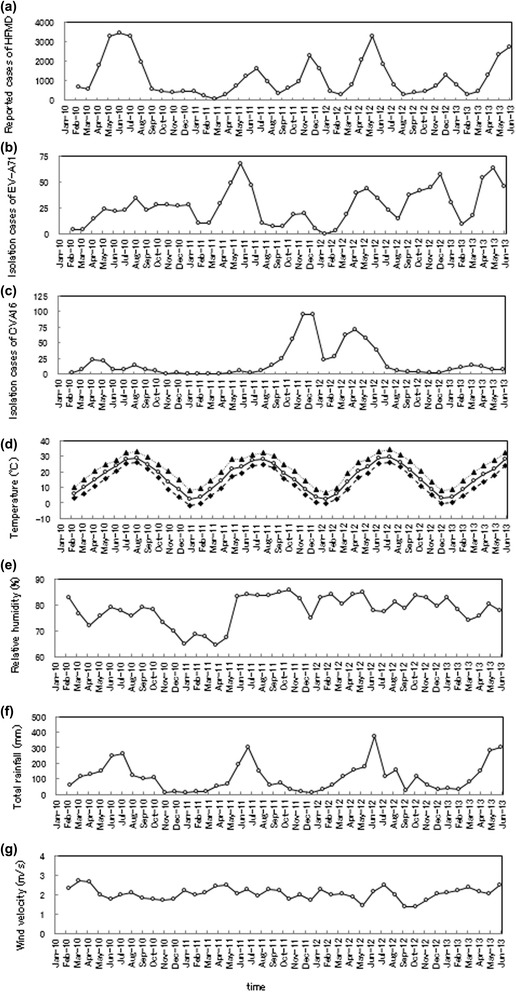
Monthly time series data for reported cases of HFMD, their pathogens, and meteorological variables. **a** HFMD, **b** EV-A71, **c** CVA16, **d** T{A}, T{M}, and T{m}, **e** RH, **f** RF, and **g** WV

The monthly time series data used in this study are illustrated in Fig. 4. For the HFMD data (Fig. 4a), two peaks occurred in a 1-year cycle, one in a summer month (June in 2010, July in 2011, and May in 2012) and the other in a winter month (December in 2011 and 2012). This bimodal cycle was also clearly observed in cases of EV-A71 infection in 2011 and 2012 (Fig. 4b), although it was not evident in 2010. Large peaks in the number of CVA16 infections (Fig. 4c) were observed in 2011 (November and December) and 2012 (April). The temporal patterns of T{A}, T{M}, and T{m} (Fig. 4d) showed large peaks in a summer month (August) in the annual cycle. The temporal pattern of RH (Fig. 4e) indicated a decreasing trend from approximately 85 % at the beginning of 2010 to approximately 65 % by May 2011. Thereafter, the temporal pattern of RH increased to approximately 80 %, and then remained relatively constant at around 80 %. The time series data for RF (Fig. 4f) indicated a large peak in the summer months of the annual cycle (June in 2010 and 2011, and May–July in 2012). However, as for RH (Fig. 4e), no seasonal pattern in the data for WV (Fig. 4g) was obvious at first glance.

### Spectral analysis and LSF analysis

**Fig. 5:**
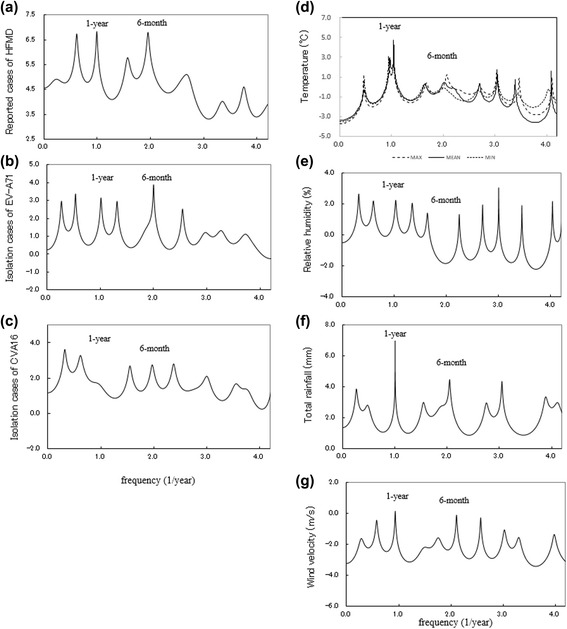
Power spectral densities of the monthly time series data. **a** HFMD, **b** EV-A71, **c** CVA16, **d** T{A}, T{M}, and T{m}, **e** RH, **f** RF, and **g** WV
(ii) *Assignment of fundamental modes:* To obtain the optimum LSF curve, we assigned the fundamental modes constructing the underlying variation in Eq. (1). In the present study, we investigated the contributions of 10 MEM-estimated periods to the LSF curve. We then calculated the SD of the residual time series with the variation in *N*_*p*_. For the HFMD data, for example in Fig. 6, the values of SD were plotted against *N*_*p*_. The figure shows inflection points at 6–8 modes. We separated the contributions of the 10 periods into two parts: the underlying variation and the fluctuating part, as described in Eq. (1). For the HFMD data, we determined *N*_*p*_ = 5 and assigned the five periods as the fundamental modes (1.63, 1.01, 0.64, 0.51, and 0.37 years), which are listed in Table 2 with the corresponding periods and intensities (powers) of the spectral peaks. The fundamental modes for the pathogen data and the meteorological data were similarly assigned at *N*_*p*_ = 5 and are shown in Table 2. In Fig. 7, each LSF curve calculated with the fundamental modes reproduces the original time series data well. The good fit of each LSF curve to the original time series data was supported by the high values of *ρ* between the original data and the LSF curve: 0.93 for HFMD, 0.95 for EV-A71, 0.90 for CVA16, 0.99 for T{A}, T{M}, and T{m}, 0.94 for RH, 0.93 for RF, and 0.82 for WV. Thus, the fundamental modes assigned to the results of the MEM spectral analysis for each set of time series data (Fig. 5, Table 2) were confirmed as appropriate.

(i) *MEM spectral analysis:* The MEM-PSDs for the time series data (Fig. 4) are shown in Fig. 5. For all PSDs, except that of CVA16 (Fig. 5c), prominent spectral peaks were observed at *f* = 1.0 (= *f*_1_), corresponding to a 1.0-year period.

**Fig. 6:**
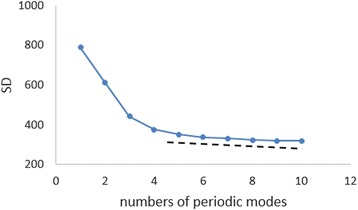
Contributions of periodic modes to the LSF curve of HFMD data

**Table 2:** Characteristics of the fundamental modes of monthly data for HFMD cases, pathogens, and meteorological variables

Variables	*f* (1/year)	Period (year)	Power
Monthly number of HFMD cases	0.61	1.63	243468.60
	0.99	1.01	200329.10
	1.57	0.64	65433.80
	1.95	0.51	336891.30
	2.68	0.37	31004.70
Monthly number of EV-A71 identification	0.54	1.86	49.84
	1.02	0.99	35.30
	1.32	0.76	27.71
	2.00	0.50	125.71
	2.55	0.39	12.41
Monthly number of CVA16 identification	0.62	1.61	200.24
	1.55	0.64	37.05
	0.62	1.61	200.24
	1.55	0.64	37.05
	1.98	0.51	46.49
	2.38	0.42	54.45
	3.00	0.33	24.37
T{A} (°C)	0.97	1.03	12.89
	1.05	0.96	62.21
	1.65	0.61	0.18
	2.02	0.50	0.34
	3.04	0.33	0.39
T{M} (°C)	0.96	1.05	4.96
	1.04	0.96	7.72
	1.68	0.60	0.68
	2.07	0.48	1.46
	3.03	0.33	1.02
T{M} (°C)	0.95	1.06	5.44
	1.04	0.96	7.73
	1.63	0.61	0.53
	2.00	0.50	0.54
	3.05	0.33	0.68
RH (%)	0.32	3.12	4.89
	0.60	1.66	3.59
	1.03	0.97	3.45
	1.35	0.74	3.00
	3.00	0.33	2.05
RF (mm)	0.26	3.81	22.80
	1.00	1.00	103.40
	2.05	0.49	37.20
	3.05	0.33	28.80
	5.15	0.19	21.40
WV (m/s)	0.58	1.72	0.16
	0.94	1.06	0.19
	2.11	0.47	0.19
	2.58	0.39	0.14
	3.03	0.33	0.09

**Fig. 7:**
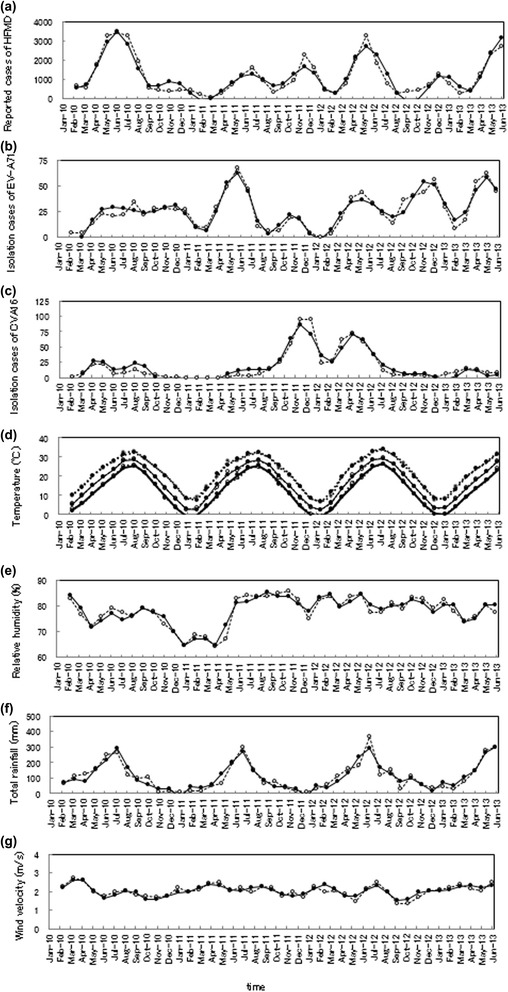
Comparison of the LSF curves calculated for the fundamental modes with the original data. LSF curve, solid line; original data, dashed line. **a** HFMD, **b** EV-A71, **c** CVA16, **d** T{A}, T{M}, and T{m}, **e** RH, **f** RF, and **g** WV

### Prediction analysis

**Fig. 8:**
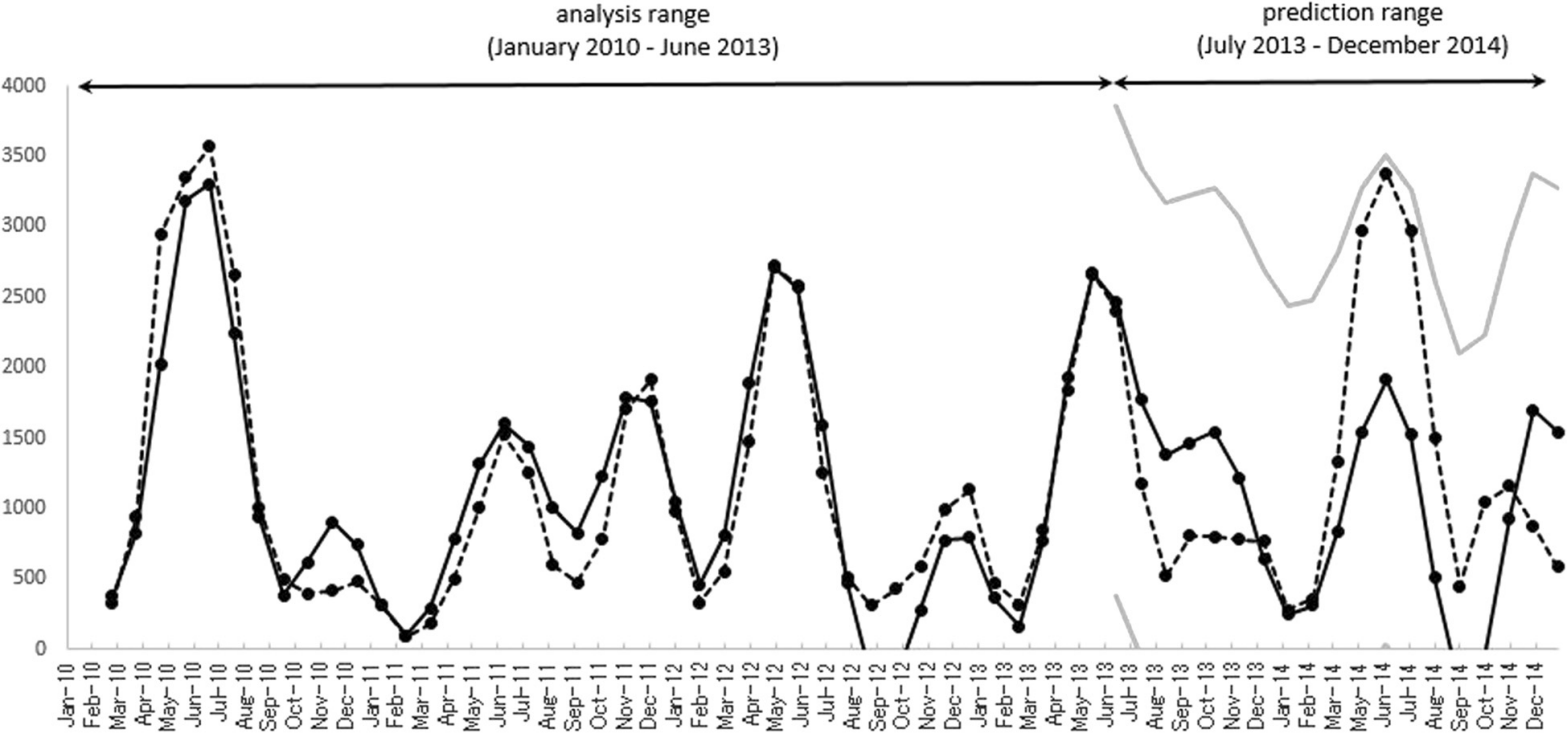
Comparison of the optimum LSF curve for the HFMD data in the prediction range. LSF curve, solid line; original data, dashed line; gray lines, 95 % confidence

The optimum LSF curve for HFMD, calculated with the five fundamental modes (Table 2), was extended from the analysis range (January 2010–June 2013) to the prediction range (July 2013–December 2014) and the results are shown in Fig. 8. The LSF curve in the prediction range reproduced the position of the peak in autumn 2013 and that in spring 2014 fairly well. The LSF curve in the prediction range (July 2013–December 2014) lies within the 95 % confidence interval, reproducing the underlying variation in the original data well.

### Bimodal cycles of HFMD data and pathogen data

It is noteworthy that the dominant spectral lines for HFMD, EV-A71, and CVA16 (Fig. 5a, b, and c, respectively) were observed at *f* = 0.5, corresponding to a 6-month period, resulting from the bimodal cycles observed in the HFMD and EV-A71 data in 2011 and 2012 (Fig. 4a and b, respectively) and in the CVA16 data in 2011–2012 (Fig. 4c).

**Fig. 9:**
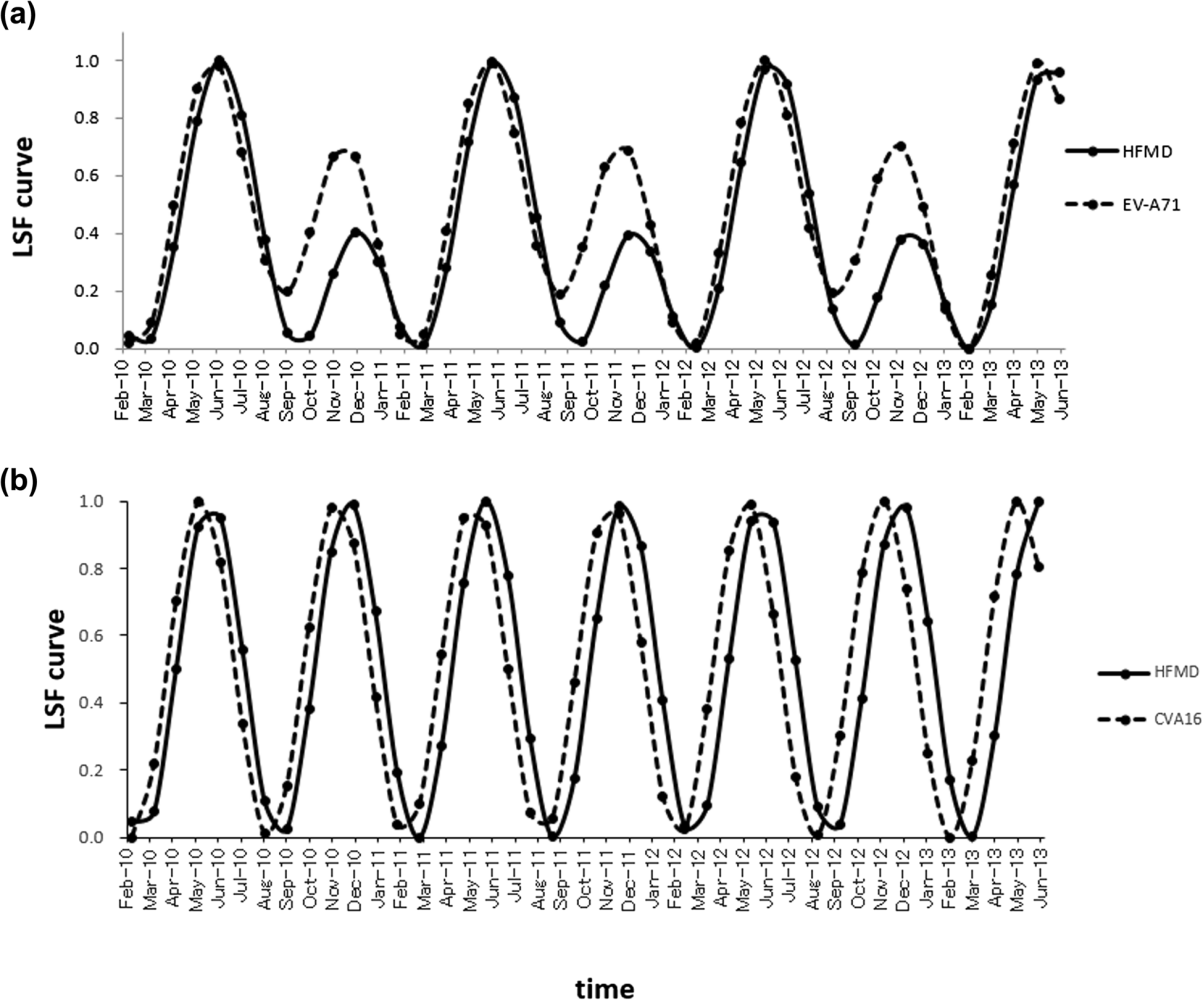
Normalized LSF curves. **a** The curves for HFMD (*solid line*) and EV-A71 (*dashed line*) calculated with the 1-year and 6-month cycles, and (**b**) the curves for HFMD (*solid line*) and CVA16 (*dashed line*) calculated with the 6-month cycle

The LSF curves for HFMD and EV-A71 were calculated with the 1-year and 6-month cycles, which were clearly observed in the PSDs (Fig. 5a and b, respectively). The LSF curves obtained were normalized in amplitude and overlapped, as shown in Fig. 9a. The peak months on the LSF curves for the HFMD and EV-A71 data during 2010–2012 were mutually consistent, whereas the peak month on the LSF curve for HFMD in 2013 (June) was delayed by 1 month relative to that for EV-A71 (May). The value of *ρ* between the LSF curve for HFMD and that for EV-A71 was high (0.90).

Similarly, the LSF curves for HFMD and CVA16 were calculated with the 6-month cycle clearly observed in the PSDs (Fig. 5a and c, respectively), and the results obtained are shown in Fig. 9b. The peak months on the LSF curve for HFMD in 2010 (June and December), 2011 (June), and 2013 (June) were delayed by 1 month relative to those for CVA16. The other peak months on both LSF curves were mutually consistent. The value of *ρ* between the LSF curve for HFMD and that for CVA16 was high (0.65).

### Correlations between EV-A71 infection and meteorological variables

The values of *ρ* between the pathogen data (EV-A71 and CVA16) and the meteorological variables are listed in Table 3. EV-A71 infections were positively associated with T{A}, T{M}, T{m}, and RF, and negatively associated with RH and WV. Of these variables, T{A}, T{M}, T{m}, and RF showed strong mutual associations with high *ρ* values ranging from 0.33 to 0.37. In contrast, RH, RF, and WV were not significantly associated with EV-A71 infections.

**Table 3:** Spearman’s correlation coefficients for monthly data on pathogens and meteorological variables

Variables	EV-A71	CVA16
T{A} (°C)	0.34^*^	0.19
T{M} (°C)	0.37^*^	0.16
T{m} (°C)	0.34^*^	0.20
RH (%)	−0.07	0.35^*^
RF (mm)	0.33^*^	0.23
WV (m/s)	−0.16	−0.03

^*^ *P* < 0.05;

**Fig. 10:**
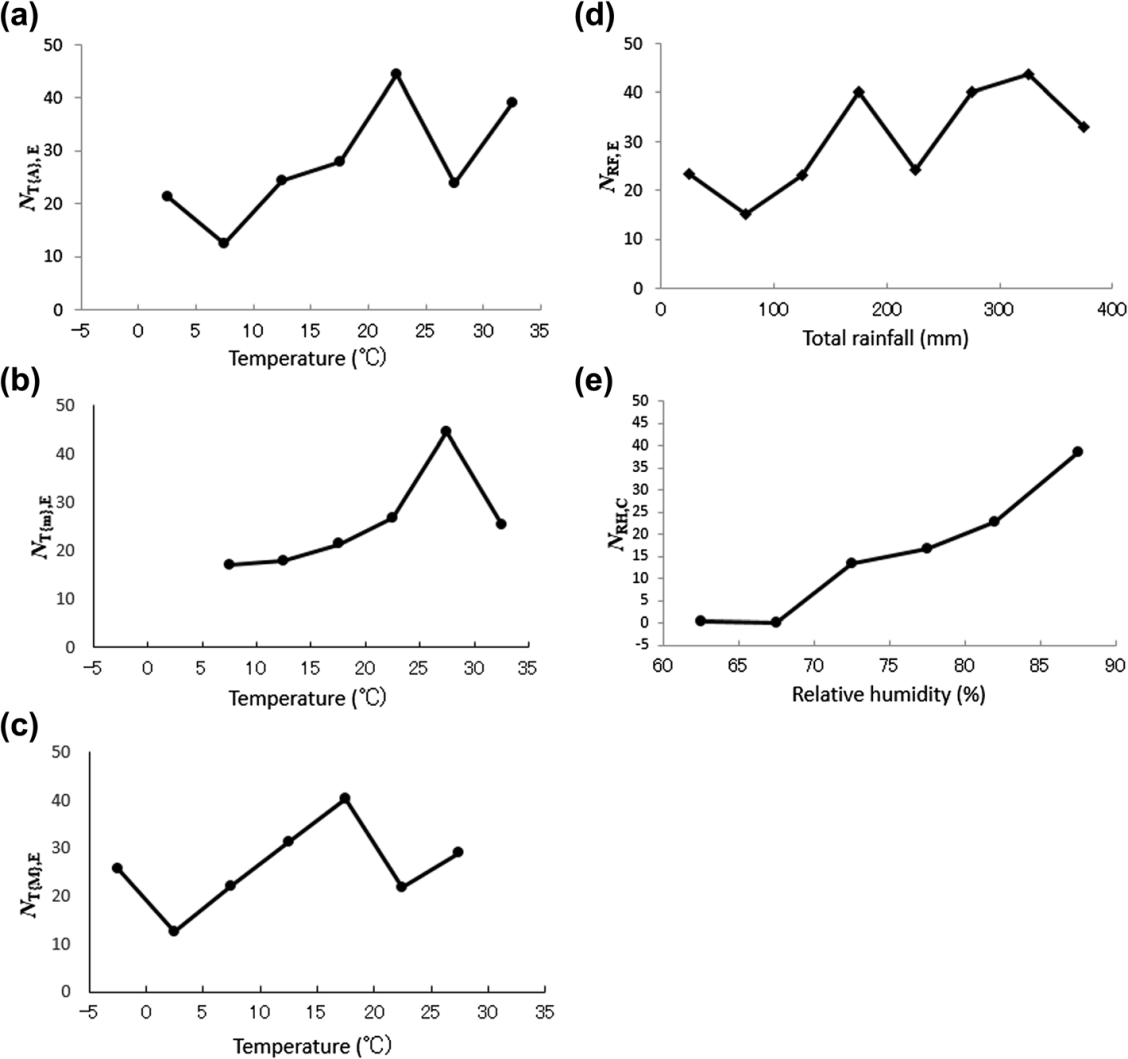
Occurrence of EV-A71 and CVA16 infections and meteorological variables. **a**–**d** Average EV-A71 infection occurrence (*N*_T{A},E_, *N*_T{M},E_, *N*_T{m},E_, and *N*_RF,E_) was defined as the average number of EV-A71 infections observed during a 1-month period for a given domain of T{A}, T{M}, T{m}, and RF. **e** The average CVA16 infection occurrence (*N*_RH,C_) was defined as the average number of CVA16 infections observed during a 1-month period for a given domain of RH

Based on the results for EV-A71 shown in Table 3, we investigated *N*_T{A},E_, *N*_T{M},E_, *N*_T{m},E_, and *N*_RF,E_ (Eq. (3))*.* The results obtained are shown in Fig. 10. In the case of T{A} (Fig. 10a), the value for *N*_T{A},E_ was relatively high when 10 °C ≤ *Temp* < 15 °C and 15 °C ≤ *Temp* < 20 °C, and peaked when 20 °C ≤ *Temp* < 25 °C. The value of *N*_T{A},E_ became small when 25 °C ≤ *Temp* < 30 °C, but increased again when 30 °C ≤ *Temp* < 35 °C.

The inverse V-shaped relationship between the *N*_T{A},E_ values against *Temp* (Fig. 10a) was also observed for *N*_T{M},E_, with a peak when 25 °C ≤ *Temp* < 30 °C (Fig. 10b), and for *N*_T{m},E_, with a peak when 15 °C ≤ *Temp* < 20 °C (Fig. 10c), corresponding to before and after the peak of *N*_T{A},E_ when 20 °C ≤ *Temp* < 25 °C (Fig. 10a). This result is consistent with the following two facts: (i) the time series data for T{A}, T{M}, and T{m} (Fig. 4d) oscillate in the same phase; and (ii) the differences between the mean values for T{A} (16.5 °C; Table 1) and T{M} (21.4 °C; Table 1) and between the mean values for T{A} (16.5 °C; Table 1) and T{m} (12.5 °C) are approximately 5.0 °C.

For the total rainfall (Fig. 10d), the value for *N*_RF,E_ was relatively high when 150 mm ≤ *r* < 200 mm and 250 mm ≤ *r* < 400 mm (*r*, total rainfall [mm]).

### Correlations between CVA16 and meteorological variables

RH was a strongly associated with CVA16 infections (*ρ =* 0.35; Table 3). Therefore, we investigated *N*_RH,C_, and the results are shown in Fig. 10e. The pattern shows a positive slope with respect to RH. However, T{A}, T{M}, T{m}, RF, and WV were not significantly associated with CVA16 infections (Table 3).

## Discussion

In this study, we found that the HFMD infections occurring in Wuhan showed two seasonal peaks, in summer (June) and winter (November or December). The LSF curves shown in Fig. 7 suggest that the bimodal seasonal peaks in the HFMD epidemics are attributable to EV-A71 and CVA16 epidemics. The following factors may explain the bimodal seasonal peaks in the EV-A71 and CVA16 epidemics in Wuhan (Fig. 4b): (i) the association between EV-A71 and CVA16 infections and meteorological variables; and (ii) the environmental conditions in Wuhan.

(i) *Association between EV-A71 and CVA16 infections and meteorological variables.* The results shown in Fig. 10 support the results of Chang et al. [25], who found that cases of HFMD were reported in Taiwan at temperatures of 13–26 °C, the temperature range in which the EV-A71 virus is activated, and decreased at temperatures lower than 13 °C or higher than 26 °C. In Wuhan, where the temperature falls below 15 °C during autumn-winter and exceeds 25 °C in summer, the occurrence of HFMD epidemics is bimodal (Fig. 4a). This is similar to a previous finding in Guangzhou, China [12], where the association between the incidence of HFMD and temperature increased rapidly below 25 °C but flattened above 25 °C.

However, the results shown in Fig. 10a indicate that the low value of *N*_T{A},E_ when 25 °C ≤ *Temp* < 30 °C returns to a high value when 30 °C ≤ *Temp* < 35 °C, which differs from the infections recorded in Taiwan [25] and Guangzhou, China [12]. This large value for *N*_T{A},E_ in Wuhan when 30 °C ≤ *Temp* < 35 °C was recorded on only one isolated occasion in July 2010 (Fig. 4b). To understand the correlation between EV-A71 infection and temperature in Wuhan in more detail, further surveillance data for EV-A71 (including data on HFMD) and other pathogens will be required. The findings of this study show that when the temperature is between 15 and 25 °C in Wuhan, public-health authorities should prepare fully to respond to an epidemic of HFMD, including increasing access to health-care resources, the distribution of scientific knowledge to the public, medical staff and public health personnel, the availability of essential medical equipment, active disease surveillance, and the design of other more-specific control measures to mitigate the risk of disease transmission.

Our finding of a positive correlation between the reported cases of EV-A71 infections and rainfall (Fig. 10d) is supported by a previous study that demonstrated that some tropical and subtropical countries experienced more outbreaks in the rainy season [26]. The large values for *N*_RF,E_ when 250 mm ≤ *r* < 400 mm (Fig. 10d) are consistent with the peak rainfall during the monsoon, which brought large amounts of rain in June 2010, June 2011, June 2012, and May–June 2013 (Fig. 4f). The large values of *N*_RF,E_ when 150 mm ≤ *r* < 200 mm correspond to the relatively high values for rainfall before and after the monsoons in April–May and July–August (Fig. 4f).

(ii) *Environmental conditions in Wuhan.* The winter peak in HFMD, which occurs after the first peak in summer, is probably attributable to disease transmission from the patients who formed the first peak because EV-A71 persists in the environment [27]. EV-A71 can be found in an infected person’s feces for several weeks after the onset of symptoms, and possibly remains for days or weeks on materials in domestic and institutional environments [28, 29]. The high population density in Wuhan could also increase the disease transmission rate and the likelihood of outbreaks.

The strong correlation between RH and CVA16 infections (Fig. 10e) may explain the fairly large numbers of CVA16 infections in the winter of 2011 and the spring of 2012, with very few cases in other years (Fig. 4c), although there has been no convincing explanation of these annual fluctuations in CVA16 infections. The annual fluctuations in disease have been interpreted in terms of many factors, including meteorological factors, host susceptibility, and changing contact rates between susceptible and infectious individuals [30]. This organizational process has been investigated with the susceptible/exposed/infective/recovered (SEIR) model, which is described with nonlinear differential equations [31, 32], but no definite conclusions regarding CVA16 infections have yet been drawn.

We found no statistically significant association between WV and either EV-A71 or CVA16. This result is inconsistent with a Hong Kong study [33] for the period 1981–2010, when WV was reported to be 3.1 m/s, which was greater than the average wind speed in Wuhan during the present study period (2.1 m/s; Table 1). It is possible that there is a threshold effect of wind speed, which is not exceeded in Wuhan.

The prevalent month/week of the seasonal cycle of HFMD incidence has attracted the attention of researchers in the hope of predicting disease outbreaks [9–13]. To investigate the seasonality of the disease incidence, some studies have used time series analyses [9–12]. One of the important approaches used with time series is the autoregressive model, which is a special case of the linear filter model, and includes sophisticated versions, such as the autoregressive moving-average model and the seasonal autoregressive integrated moving-average model [9, 34]. In the present study, we applied our prediction analysis method to the HFMD data (Fig. 8). The present method is based on the most traditional method of prediction analysis, which uses an extrapolation curve corresponding to the underlying variations of the time series data, *X*(t) (Eq. (2)) in future. The reproducibility of the HFMD data is considered to arise because the fundamental modes constructing *X*(t) (Table 2) were well assigned by the MEM spectral analysis and reconstruct the periodic structure of the underlying variation in the data in the prediction range (Fig. 8). We anticipate that the present method of time series analysis using an MEM spectral analysis and LSM will allow the further development of prediction analyses for HFMD epidemics.

A limitation of this study was that we used monthly pathology data for EV-A71 and CVA16 rather than daily or weekly data, because monthly measures are the minimum unit of measurement released by the CISDCP. Further studies using daily or weekly data are required in the future. Another limitation was that the percentage of laboratory confirmation was low (< 5 %), because the purpose of testing samples from HFMD cases is to determine the predominant virus circulating in Wuhan, rather than to identify further patients with the disease.

## Conclusion

The results of our study indicate that in Wuhan, EV-A71-based HFMD infections correlate strongly with the average, maximum, and minimum temperatures and total rainfall, and that CVA16-based HFMD infections correlate strongly with relative humidity.

The Intergovernmental Panel on Climate Change Third Assessment Report states that “changes in climate that will affect potential transmission of infectious diseases include temperature, humidity, altered rainfall, and sea-level rise” [35]. EV-A71 and CVA16 lack a thermostatic mechanism, and their reproduction and survival rates are strongly affected by fluctuations in **t**emperature, as are those of other viruses, parasites, and bacteria [36, 37]. Therefore, the effects of meteorological variables on the epidemiology of EV-A71 and CVA16 must be investigated to control HFMD, as in this study.

## Additional files

Additional file 1:
**Meteorological parameters used for examining the relationship with the monthly EV-A71 and CVA16 data.** (XLSX 10 kb) Additional file 2:
**MEM spectral analysis.** (DOCX 17 kb)

## Abbreviations

HFMD: Hand, foot, and mouth disease
EV-A71: Enterovirus A71
CVA16: Coxsackievirus A16
T{A}: Average temperature
T{M}: Maximum temperature
T{m}: Minimum temperature
RH: Relative humidity
RF: Rainfall
WV: Wind velocity
MEM: Maximum entropy method
LSF: Least squares fitting
LSM: Least squares method
PSD: Power spectral density
SD: Standard deviation
