# Supplementary material for: Time series analysis of reported cases of hand, foot, and mouth disease from 2010 to 2013 in Wuhan, China
Source: BMC Infect Dis. 2015 Nov 3;15:495. doi: 10.1186/s12879-015-1233-0 (PMC4630926; doi:10.1186/s12879-015-1233-0)
Supplement: Additional file 2: — MEM spectral analysis. (DOCX 17 kb) [file 12879_2015_1233_MOESM2_ESM.docx]

# **Additional file 2**

**MEM spectral analysis**

MEM power spectral density (MEM-PSD) *P*( *f* ) (where *f* represents frequency) for the time series with equal sampling interval ∆*t*, can be expressed by

$$P\left(f\right)=\frac{P_{m}\Delta t}{\left|1+\sum_{k=-m}^{m} \gamma_{m.k}\exp\left[-i2\pi fk\Delta t\right]\right|^{2}},$$

where the value of *P_m_* is the output power of a prediction-error filter of order *m* and *𝛾_m, k_* is the corresponding filter order. The value of the MEM-estimated period of the *n*th peak component *T_n_* (=1/*f_n_*; where *f_n_* is the frequency of the *n*th peak component) can be determined by the positions of the peaks in the MEM-PSD.
